# Editorial: Anakoinosis for promoting tumor tissue editing: Novel therapeutic opportunities for establishing clinically relevant tumor control by targeting tumor plasticity

**DOI:** 10.3389/fonc.2022.1005381

**Published:** 2022-09-13

**Authors:** Daniel Heudobler, Lina Ghibelli, Albrecht Reichle

**Affiliations:** ^1^ Department of Internal Medicine III, Hematology and Oncology, University Hospital Regensburg, Regensburg, Germany; ^2^ Bavarian Cancer Research Center (BZKF), University Hospital Regensburg, Regensburg, Germany; ^3^ Department of Biology, University of Rome Tor Vergata, Rome, Italy

**Keywords:** tumor tissue editing, tumor plasticity, homeostasis, anakoinosis, differentiation, immunosurveillance

Modern systemic tumor therapies aiming at elimination of cancer cells refer to the observation that tumor evolution is triggered by acquired oncogenic events and that those acquired aberrations are available as targets for eradicating tumor cells. Alternatively, epitopes on tumor cells serve as target structures. Besides chemotherapy, these treatment strategies dramatically improved outcome, also in relapsed or refractory metastatic tumors.

A major obstacle for long-term tumor control, however, remains tumor cell heterogeneity and the oncogenic role of tumor therapy comprising cancer repopulation and acquired tumor cell resistance (CRAC) including promotion of metastases (M-CRAC) (1–9). M-CRAC is commonly addressed by empirical maintenance and consolidation therapies.

## Tissue editing

In contrast, tumor tissue editing refers on the observation that tumor tissue phenotypes can be made ‘recessive’, also triggered by non-tumor cell autonomous, communicatively mediated, tumor stroma-induced functions. Phenotypic ‘recessivity’ is characterized by a huge developmental plasticity that facilitates tumor evolution and adaption without the need of genetic changes (Lüke et al.) Klicken oder tippen Sie hier, um Text einzugeben. But, tumor plasticity also opens a therapeutic window by tumor tissue editing, the possibility to induce completely novel tumor phenotypes by introducing therapeutically relevant editing techniques that redirect hallmarks of cancer into biologic hallmarks attenuating tumor growth or even facilitate tumor cell death as prerequisite for continuous complete remission (cCR), also in relapsed or refractory neoplasias (10, 11). Aims of tumor tissue editing strategies may be differentiation induction in hematologic neoplasia or solid tumors, reconstitution of immunosurveillance, control of tumor-associated inflammation, inhibition of M-CRAC etc. (Lüke et al., Bar-Hai and Ishay-Ronen) Klicken oder tippen Sie hier, um Text einzugeben.

## Engaging tumor tissue’s plasticity as therapeutic target

Dysregulated homeostasis as basis for tumor plasticity has been proven in clinical trials as a major therapeutic starting point for tumor tissue editing. Important examples are given by contributions to the Research Topic:

* Dysregulated homeostatic processes stimulate post-chemotherapy tumor progression by increasing, but reversible expression of CRIPTO in lung cancer stem cells (Francescangeli et al.) Klicken oder tippen Sie hier, um Text einzugeben.. Shear stress in ovarian cancer cells decisively determines chemosensitivity (Bileck et al.) Klicken oder tippen Sie hier, um Text einzugeben.

* Therapeutically engaging immune cells for improving immunosurveillance leads to impressive clinical results as shown in metastatic melanoma (Tang et al.) Klicken oder tippen Sie hier, um Text einzugeben.

* The activation of tumor-directed T-cells with pioglitazone or metronomic chemotherapy may contribute to tumor immune response in resistant or relapsed neoplasia Klicken oder tippen Sie hier, um Text einzugeben. (Lüke et al., Fante et al.).

* The activation of NK cells by low-dose decitabine underlines dose-dependent anti-cancer immune response (Li et al.) Klicken oder tippen Sie hier, um Text einzugeben. A suggested NK cell activation via engagement of toll-like receptors following chemotherapy-induced mycotic infection seems to contribute to continuous complete remission in acute lymphoblastic leukemia despite of massively reduced induction chemotherapy (12).

* Differentiation induction in hematologic neoplasia could be demonstrated in non-acute promyelocytic leukemias (non-APL) and in animal models for breast cancer with pioglitazone and MEK inhibitor (13, 14).

On the background of multifold available, differentially designed tissue editing approaches, tumor tissue editing is still at its beginnings and more accompanying diagnostic instruments should be implemented. Umbrella trials would be an ideal platform for the further evaluation of tissue editing approaches (Lüke et al.) Klicken oder tippen Sie hier, um Text einzugeben.

## Tumor tissue editing techniques

Editing techniques use combinatory bio-regulatory active drugs, communicatively reprogramming tumor tissue functions, i.e., anakoinosis. Mono-activity in a distinct histologic tumor type is no prerequisite (Lüke et al.) Klicken oder tippen Sie hier, um Text einzugeben. The regulatory active dose may be far below the maximum tolerable dose, as exemplarily shown for decitabine or azacitidine (Li et al (15)., Klicken oder tippen Sie hier, um Text einzugeben.. Besides inhibitory drugs, like hormone receptor inhibitors, stimulatory drug activities may be integrated, such as the nuclear receptor agonist pioglitazone. Metronomic scheduling often seems to be of advantage (Fante et al.) Klicken oder tippen Sie hier, um Text einzugeben..

Among tumor tissue editing approaches are also anti-angiogenic strategies, e.g., metronomic low-dose chemotherapy, but also many successful tissue ‘engineering’ immunotherapies, modifying T-cell response, such as immune checkpoint inhibitors, bispecific antibodies, CAR T-cells as exemplified in a topic paper for metastatic melanoma (Tang et al., Fante et al.) Klicken oder tippen Sie hier, um Text einzugeben.

Multifold dysbalanced homeostatic pathways among cellular tumor compartments may be targeted by a broad repertoire of biomodulatory approaches including a wide range of quite different drugs to finally gain clinically important tumor phenotypes via tissue editing approaches, as discussed by Lüke et al.Klicken oder tippen Sie hier, um Text einzugeben.

## Novel outcome parameters

**Figure 1:**
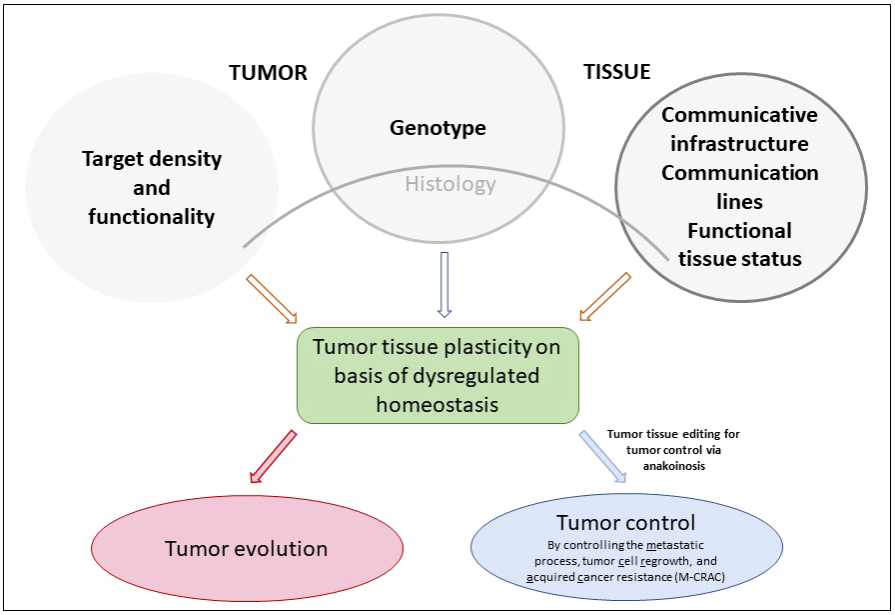
Genotype, target density and functionality, communicative infrastructure and functional status of tumor tissues determine tumor tissues’ plasticity via dysregulated homeostatic pathways that are the targets for pro-anakoinotic, concerted biomodulatory therapy approaches, designed for stopping tumor growth or for inducing continuous complete remission in the best case, just in refractory or relapsed neoplasia.

Using dysregulated tumor tissue homeostasis as therapeutic targets, multileveled reprogramming possibilities arise to therapeutically ‘normalize’ tumor tissue functions. Thereby, novel tumor tissue behaviors may be implemented in a therapeutically relevant way, such as M-CRAC control. M-CRAC control is a unique feature of a distinct tumor tissue editing technique, including metronomic chemotherapy and pioglitazone besides other metronomically applicated drugs (Lüke et al) Klicken oder tippen Sie hier, um Text einzugeben.. Apoptosis pathways become unnecessary with differentiation inducing or inflammation inhibiting therapies, while M-CRAC is tout-court bypassed, importantly, also without any additional repurposed targeted therapies (7); Klicken oder tippen Sie hier, um Text einzugeben. Bar-Hai and Ishay-Ronen, Bileck et al.) (**Figure 1** **)**.

Tissue editing approaches address genetic and molecular-genetic tumor heterogeneity at metastatic sites by exploiting the ‘recessivity’ of the tumor phenotype. Independent of the involved organ systems, metastases may be controlled for long-term, even more, cCR may be achieved in refractory or relapsed tumor disease (Lüke et al.) Klicken oder tippen Sie hier, um Text einzugeben.

## Tumor tissue editing by drug repurposing

Tumor tissue editing requires a novel understanding of tumor pathophysiology, of therapy strategies and techniques, and generates beyond the achievement of cCR in refractory/relapsed tumors highly differential outcome parameters compared to classic targeted therapy or chemotherapy. (Lüke et al.)Klicken oder tippen Sie hier, um Text einzugeben.

Multifold, quite different drugs can be synergistically used in pro-anakoinotic schedules. Synergies of targeted therapies applied at maximum tolerable doses are rare and results are dependent on the stromal context (16). In contrast, tissue editing techniques uniquely use communicative synergies that may be achieved with minimal ‘biomodulatory’ active doses. Therefore, drugs for tissue editing are repurposed if the concerted editing effect is the primary intention (Lüke et al.) Klicken oder tippen Sie hier, um Text einzugeben.

The second important therapeutic issue, as shown in clinical trials, is that drugs with no or poor monoactivity in the respective tumor disease may gain decisive activity in edited tumor tissues. Following tumor tissue editing, new targets may be presented and novel contexts for a suitable activity profile of an otherwise less efficacious targeted therapy (Lüke et al.) Klicken oder tippen Sie hier, um Text einzugeben.

An important consequence ensues from these observations: targeted therapies should be primarily tested for efficacy in differentially edited tumor tissues, as important activity profiles could be overlooked. It is not sufficient to simply test drugs by histology and expression levels of targets. The functional tumor tissue status should be added as test criterium (Lüke et al.) Klicken oder tippen Sie hier, um Text einzugeben.

## Conclusion

By describing the pathophysiologic basis of the novel therapy technique, tumor tissue editing, and related outcome parameters that are supplementing those of ‘pulsed’ therapies, the Research Topic detailed a new avenue to address and resolve therapeutic problems arising with refractory or relapsed neoplasias.

## Author contributions

All authors listed have made a substantial, direct, and intellectual contribution to the work and approved it for publication.

## Conflict of interest

The authors declare that the research was conducted in the absence of any commercial or financial relationships that could be construed as a potential conflict of interest.

## Publisher’s note

All claims expressed in this article are solely those of the authors and do not necessarily represent those of their affiliated organizations, or those of the publisher, the editors and the reviewers. Any product that may be evaluated in this article, or claim that may be made by its manufacturer, is not guaranteed or endorsed by the publisher.
